# Preimplantation genetic diagnosis for a patient with multiple endocrine neoplasia type 1: case report

**DOI:** 10.5935/1518-0557.20180010

**Published:** 2018

**Authors:** Aline DT Lima, Vanessa R Alves, Andressa R Rocha, Ana C Martinhago, Ciro Martinhago, Nilka Donadio, Artur Dzik, Mario Cavagna, Luiz H Gebrim

**Affiliations:** 1 Perola Byington Hospital - Women's Health Reference Center; 2 Chromosome Genomic Medicine

**Keywords:** Human reproduction, preimplantation genetic diagnosis, multiple endocrine neoplasia, embryo biopsy

## Abstract

Preimplantation genetic diagnosis was carried out for embryonic analysis in a patient with multiple endocrine neoplasia type 1 (MEN1). This is a rare autosomal-dominant cancer syndrome and the patients with MEN1 are characterized by the occurrence of tumors in multiple endocrine tissues, associated with germline and somatic inactivating mutations in the MEN1 gene. This case report documents a successful preimplantation genetic diagnosis (PGD) involving a couple at-risk for MEN1 syndrome, with a birth of a healthy infant. The couple underwent a cycle of controlled ovarian stimulation and intracytoplasmic sperm injection (ICSI). Embryos were biopsied at the blastocyst stage and cryopreserved; we used PCR-based DNA analysis for PGD testing. Only one of the five embryos analyzed for MEN1 syndrome was unaffected. This embryo was thawed and transferred following endometrial preparation. After positive βHCG test; clinical pregnancy was confirmed by ultrasound, and a healthy infant was born. PGD for single gene disorders has been an emerging therapeutic tool for couples who are at risk of passing a genetic disease on to their offspring.

## INTRODUCTION

Multiple endocrine neoplasia (MEN) is a rare autosomal-dominant disease characterized by the occurrence of tumors involving two or more endocrine glands in a single patient. Four major forms of MEN are recognized and referred to as types 1-4, and each one is characterized by the development of tumors within specific endocrine glands. MEN1 is characterized by the occurrence of pancreatic islet, parathyroid, and anterior pituitary tumors. MEN2, previously referred to as MEN2A, is characterized by the occurrence of medullary thyroid carcinoma (MTC) in association with pheochromocytoma and parathyroid tumors. MEN3, previously referred to as MEN2B, is characterized by the occurrence of MTC and pheochromocytoma in association with a marfanoid habitus, mucosal neuromas, medullated corneal fibers and intestinal autonomic ganglion dysfunction, leading to megacolon. MEN2 and MEN3 affect men and women equally, with an incidence of 1 for every 30,000 individuals. MEN4, which is also referred to as MENX, and it is characterized by the occurrence of parathyroid and anterior pituitary tumors in possible association with tumors of the adrenals, kidneys, and reproductive organs (Guimarães, 2007; Maia *et al*., 2005; Maia *et al*., 2002; Thakker, 2014).

MEN1 was first described in 1954 by Wermer, and it is a syndrome characterized by the combined occurrence of tumors of the pancreatic islets cells, parathyroid glands and the anterior pituitary (Hoff & Hauache, 2005). In 1988, the MEN1 locus was mapped to chromosome 11q13. It encodes a 610-amino acid protein, menin, responsible for the inhibition of many transcription factors that regulate cell cycle, cell differentiation and apoptosis (Hoff & Hauache, 2005; Pasternak, 2007). The incidence of MEN1 has been estimated to be 1 in 50,000 individuals with a reported age range of 5 to 81 years. Clinical manifestations of the disorder have developed in 80% of the patients by their fifth decade of life (Table 1) (Guimarães, 2007; Pasternak, 2007; Thakker, 2014). These clinical manifestations are related to tumor site, their secretory products, tumor size and malignant potential. In most cases, familial heterogeneity and variable penetrance are present (Guimarães, 2007; Pasternak, 2007).

**Table 1:** MEN1 syndrome - key clinical features of (adapted from Hoff, Hauache, 2005).

Syndrome	Key clinical features	Genetics
MEN1	Pituitary adenoma	Loss of function inMEN1-encoded menin protein (11q13)
	Primaryhyperparathyroidism	
	Pancreaticislettumors	
	Adrenals tumors	
	Cutaneousangiofibromas	
	Lipomas	

Depending on the extension and affected areas of the MEN 1 tumors, symptoms may include hypercalcemia leading to urolithiasis; hypoglycemia causing weight loss, mental confusion and dizziness; erectile dysfunction; infertility, amenorrhea in women, and other clinical features. The majority of MEN1 tumors are benign adenomas that compress surrounding tissues. When malignancy occurs, cancerous cells producing hormones may invade adjoining tissues or spread to distant sites (Pasternak, 2007).

Advances in molecular biology and genetics have been favoring clinical diagnosis and possible therapies. In recent years, preimplantation genetic diagnosis (PGD) enables patients who are carriers or who are affected by genetic diseases, to select unaffected embryos for transfer and increase their likelihood of having a successful pregnancy. PGD is useful in the diagnosis of a variety of genetic disorders that are caused by a known single gene mutation, X-linked diseases, number of chromosomes or structure abnormalities, such as reciprocal or Robertsonian translocations (Van der Aa *et al*., 2013; Yan *et al*., 2014). Most of these couples are fertile, but for embryo selection an *in vitro* fertilization (IVF) procedure is required (Harper & SenGupta, 2012).

The embryo should be biopsied for PGD analysis (Oliveira *et al.*, 2009). Currently, there are three potential methods according to the source of DNA collection: first or second polar body biopsy, blastomere biopsy (aspiration) from the 6- to 8-cell cleaving embryos, and trophectoderm biopsy from blastocyst on the fifth or sixth day of development (Aquino *et al.*, 2013; Yan *et al.*, 2014).

This case report documents a successful IVF-PGD involving an at-risk couple for MEN1 syndrome, with a birth of a healthy infant.

## CASE REPORT

Following a MEN1 diagnosis, the couple was referred to assisted reproductive technology (ART). They signed a free informed consent before beginning the procedure.

A 34 years old male patient, carrier of MEN1 syndrome, with a normal semen analysis according to the WHO, was enrolled for ICSI-PGD, to have an unaffected embryo.

**Figure 1:**
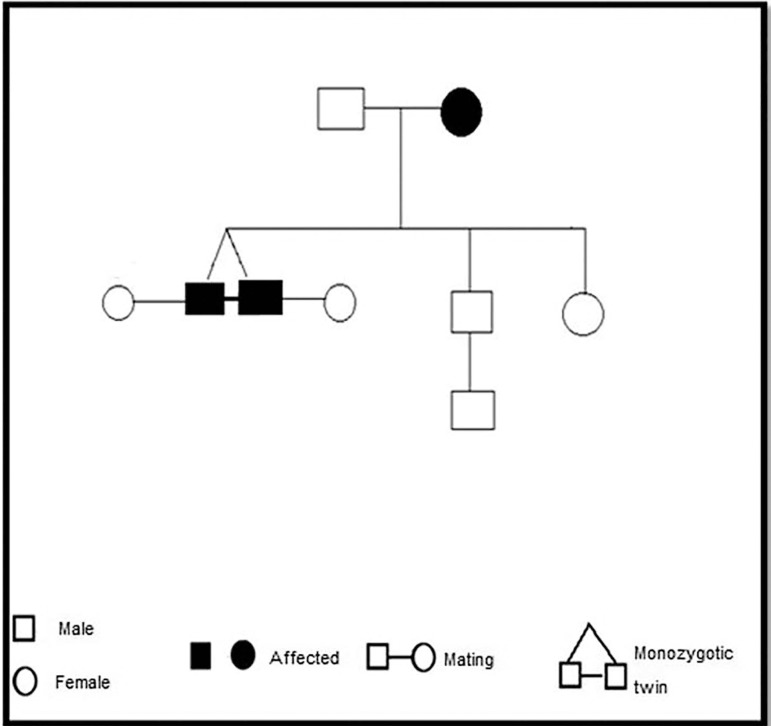
Familial pedigree.

After propaedeutic and family history (Figure 1), TMP, 32 years old female patient underwent ovarian stimulation with GnRH agonist (Triptorelin acetate 3.75mg/mL) with recombinant FSH (Alfa Follitropin 150UI+Lutropin 75UI) for 10 days. When at least one of the follicles reached ≥18 mm, 5,000UI of human chorionic gonadotrophin (hCG) were administered. Oocyte retrieval happened through transvaginal ultrasonography, performed 36-38 hours after the administration of hCG. A total of 19 oocytes were collected, and 15 mature oocytes (in metaphase II) were injected, resulting in 11 viable embryos. The zona pellucida was opened for blastomere removal using a laser on day 3 (72h). At the blastocyst stage (Day 6), five embryos were biopsied and vitrified for frozen embryo transfer after PDG results. The removed trophectoderm was transferred to microtubes containing lysis buffer. The embryo genome was amplified with the Genome Plex^®^ Whole Genome Amplification (WGA) kit. PCR was performed using specific primers to identify the MEN1 gene mutation. DNA *Sequencing* was done by capillary electrophoresis in an ABI3500 automatic sequencer, and analyzed using the Gene Mapper^®^ and Gene Marker^®^ *software*.

As seen in Table 2, PGD analysis reported that only one of the five embryos analyzed for MEN1 mutation was unaffected.

**Table 2:** Genetic analysis report

Embryo	c.650delA/p.E217GfsX7 mutation	Result
E10	Heterozygous mutation	Affected
E15	Heterozygous mutation	Affected
E7	Heterozygous mutation	Affected
E6	Heterozygous mutation	Affected
E8	No mutation	Normal

This embryo was thawed and transferred after 3 hours, following endometrial preparation with estradiol and progesterone. A positive serum βHCG result was obtained after 15 days. Clinical pregnancy was confirmed by ultrasound with gestational age of six weeks. In the 38 weeks of gestation, a boy was delivered by cesarean section, Apgar scores 8/9. Genetic analysis was done and resulted in a healthy infant, without MEN1 gene mutation.

## DISCUSSION

MEN1 mutations have inactivating power and are consistent with a tumor suppressor gene. The genetic study has some limitations due the absence of hotspots as the RET gene. As an autosomal dominant inheritance, in a patient with MEN1 syndrome, offspring has about a 50% risk of developing the disease (Guimarães, 2007; Westman, 2006). PGD was indicated due its valuable option for patients with the high risk of transmitting an inherited disease to their offspring. Blastomeric analysis is prone to misdiagnosis due to chromosomal mosaicism, technique limitations - such as contamination or amplification failure, or even high allele drop-out. Nevertheless, the accuracy is about 90% for the detection of new mutations (Yan *et al.*, 2014).

There are multiple genetic analysis and biopsy methods for PGD at different embryonic development stages. In this specific case, blastocyst biopsy and PCR were chosen to improve accuracy and specificity for single gene mutation (Thornhill *et al.*, 2005; Handyside, 2013).

Blastocyst biopsy has been widely applied in PGD because it enables the analysis of an active embryonic genome. In addition, as an advantage compared to other techniques, blastocyst biopsy provides an adequate biological sample with low risk to the embryo. Patient age, number of viable embryos and their quality were also considered.

Despite the blastocyst biopsy advantages, in cases of advanced maternal age, there maybe be fewer oocytes compared to younger patients. Therefore, less available embryos or even none blastocyst for biopsy (Aquino *et al.*, 2013). Couples who are at risk of passing a genetic disease on should be advised to not postpone a pregnancy, to produce more viable embryos to select an unaffected one.

## CONCLUSION

In the present study, we report a successful birth of a healthy infant following trophectoderm biopsy from blastocysts for MEN1 syndrome. Preimplantation genetic diagnosis is an outstanding embryonic diagnostic tool before embryo transfer. Besides improving ICSI outcomes, PGD by PCR-based DNA analysis also benefits couples at risk of passing a genetic disease on to their offspring, enabling the selection of unaffected embryos.
